# The difference of affect improvement effect of music intervention in aerobic exercise at different time periods

**DOI:** 10.3389/fphys.2024.1341351

**Published:** 2024-04-29

**Authors:** Li Lu, Meng Tao, Jingchuan Gao, Mengru Gao, Houwei Zhu, Xiaolong He

**Affiliations:** ^1^ Department of Physical Education and Health Science, Zhejiang Normal University, Jinhua, China; ^2^ School of Exercise and Health, Shanghai University of Sport, Shanghai, China

**Keywords:** aerobic power cycling, music, affect, heart rate variability, exercise

## Abstract

**Objectives:** A randomized controlled experimental design that combines exercise and music intervention was adopted in this study to verify whether this approach could help improve human affect. The differences in the effect of music listening on affective improvement were compared in four different periods: before, during, and after aerobic power cycling exercise and the whole exercise course.

**Method:** A total of 140 subjects aged 19–30 years (average age: 23.6 years) were recruited and randomly divided into four music intervention groups, namely, the pre-exercise, during-exercise, post-exercise, and the whole-course groups. The subjects’ demographic and sociological variables and daily physical activities were collected using questionnaires. Individual factors, such as the subjects’ noise sensitivity, personality traits, and degree of learning burnout, were collected via scale scoring. A laboratory in Zhejiang Normal University was selected as the experimental site. The testing procedure can be summarized as follows. In a quiet environment, the subjects were asked to sit quietly for 5 min after completing a preparation work, and then they were informed to take a pre-test. The four subject groups wore headphones and completed 20 min of aerobic cycling (i.e., 7 min of moderate-intensity cycling [50%*HRR + RHR] + 6 min of low-intensity interval cycling [30%*HRR + RHR] + 7 min of moderate-intensity cycling [50%*HRR + RHR] after returning to a calm state (no less than 20 min) for post-testing. The affect improvement indicators (dependent variables) collected in the field included blood pressure (BP), positive/negative affect, and heart rate variability indicators (RMSSD, SDNN, and LF/HF).

**Results:** 1) Significant differences were found in the participants’ systolic BP (SBP) indices and the effect of improvement of the positive affect during the exercise–music intervention among the four groups at different durations for the same exercise intensity (*F* = 2.379, *p* = 0.030, *ɳp* ^*2*^ = 0.058; *F* = 2.451, *p* = 0.043, *ɳp* ^*2*^ = 0.091). 2) Music intervention for individuals during exercise contribute more to the reduction of SBP than the other three time periods (*F* = 3.170, *p* = 0.047, *ɳp* ^*2*^ = 0.068). Improvement in the participants’ negativity affective score was also better during exercise, and it was significantly different than the other three time periods (*F* = 5.516, *p* = 0.006, *ɳp* ^*2*^ = 0.113). No significant differences were found in the improvement effects of the other effective indicators for the four periods.

**Conclusion:** Exercise combined with music intervention has a facilitative effect on human affect improvement, and listening to music during exercise has a better impact on affective improvement than music interventions at the other periods. When people perform physical activities, listening to music during exercise positively affects the progress effect among them.

## 1 The statement of relevance

An extensive search of the empirical research literature reported internationally has revealed that most exercise–music interventions aimed at improving human mental health or real-time emotions combine “exercise” and “music” simultaneously or have focused on a single-phase activity during the time of auditory music stimulation. However, studies are rare in terms of comparing the effects of music auditory interventions on human mental health improvement or real-time affect at different times of the exercise process (e.g., before, during, and after exercise or throughout the exercise process). Aiming to address the research gap, this study adopts a randomized controlled trial design, giving the same auditory music stimuli before, during, and after aerobic power cycling and throughout the exercise process, to compare the effect of each group on affect improvement. The findings of this research can provide a reference for future combined exercise–music intervention strategies.

## 2 Introduction

Music therapy is a therapeutic technique that incorporates the knowledge of medicine, psychology, music aesthetics, physics, and other disciplines, in which music is used to change human behavior, emotions, and physiological functions.In recent years, music therapy has been widely used around the world to relieve stress and improve mood in a variety of clinical populations (Chanda and Levitin, 2013; Koelsch, 2015; Mehr et al., 2019; De Witte et al., 2020). As a highly complex sound medium, music involves the listener’s hearing, attention, perception, analysis of sound patterns, memory, and emotional experience, and is the general stimulus that evokes the highest level of personal experience (Koelsch et al., 2005). The integrative cognitive processing properties of music play a very important role in human life, especially in the five areas of emotion regulation, language facilitation, communication facilitation, motor facilitation and cognitive regulation (Harikumar et al., 2006). In addition, music can reduce individual stress by decreasing physiological arousal, as evidenced by lower cortisol levels, lower heart rate, and lower mean arterial pressure (Kreutz et al., 2012; Linnemann et al., 2015; Burrai et al., 2016; Koelsch et al., 2016). Music also reduces negative emotions and feelings such as subjective apprehension, state anxiety, restlessness or nervousness, and increases positive emotions and emotional feelings (Levitin, 2009; Moore, 2013; Salimpoor et al., 2013; Zatorre, 2015). In addition, listening to music stimulates the brain to trigger feelings of pleasure, enriches the functioning of neural networks and strengthens the body’s immunity.Therefore, this type of therapy has been commonly used to treat individuals with mental, cognitive, and physical disorders (Agres et al., 2021; Bainbridge et al., 2021).

Music intervention, as an adjunct activity to exercise, has been widely used in music therapy, and this exercise–music intervention combination has been reported to help individuals recover from post-exercise exercise fatigue and improve their positive affect (Chair et al., 2021). Research findings have shown that music interventions prior to exercise can have the effect of bringing individuals to a state of arousal or relaxation. Eliakim et al. (2007) conducted a study on the effect of musical stimulation during warm-up exercise on the aerobic capacity produced by youth volleyball players at the national level, and the results showed that after a 10-min warm-up exercise in a musical environment, the athletes’ anaerobic thresholds increased and their performance was higher than that of the athletes who did not listen to music during the warm-up exercise (Eliakim et al., 2007; Karageorghis et al., 2018a). A study by Hutchinson et al. (2018) and others combined music and exercise, leading to the conclusion that people who listen to music are able to maintain a higher intensity of exercise while still feeling and feeling good compared to those who do not listen to music, and that when music is used during exercise, it can elicit a positive affect in the individual and distract the exerciser or athlete from unpleasant feelings associated with physical strength and fatigue of attention (Hallgren, et al., 2010; Raichlen et al., 2012; Heggelund et al., 2014). These effects include improved individual strength and energy output efficiency, increased endurance, and increased productivity (Atkinson et al., 2004; Hutchinson et al., 2011; Terry et al., 2012; Lee and Kimerly, 2016; Karageorghis et al., 2018b).

Modern music therapy mainly consists of passive music therapy (listening to music) and active music therapy (from participating in musical activities to regularly listening to music). Some studies have shown that in people who suffer from depression, they tend to show a decrease in NE, E, 5- HT, DA, etc., and performing exercise can promote the secretion of NE, E, and 5-hydroxytryptamine (Choi et al., 2018). Therefore, it is theorized that physical activity stimulates the secretion of neurotransmitters, which in turn contributes to the improvement of an individual’s depressive mood. It has been suggested that stimulation through a single sensory modality, such as visual or auditory, as well as physical exercise have the ability to alleviate or exacerbate depressive symptoms as well as improve mood, depending on several parameters such as the intensity, frequency, duration, and quality of these stimuli (Takano et al., 2022).Meanwhile, music therapy helps people to alleviate the dullness, monotony, and fatigue of prolonged exercise (Bigliassi et al., 2017).

Music therapy can also attract attention, trigger a range of emotions, alter or regulate affect, evoke memories, increase workload, improve arousal, induce high-functioning states, reduce inhibitions, and encourage rhythmic movement-all purposes that have considerable application in the field of exercise (Chanauria et al., 2019; Shen et al., 2019). The facilitating effect of music on the functioning of an individual’s organism is more pronounced when music improves exercise performance by delaying fatigue or increasing the individual’s positive affective experience. Often, this facilitating effect results in the exercise subject producing higher than expected levels of endurance, strength, productivity, or power. In this sense, music can be considered as a more appropriate intervention to improve an individual’s motor performance (Zoormand, 2023). With the development of cognitive neuroscience techniques in recent years, the study of brain mechanisms of music as a means of improving human affect has been further developed, humans have the ability to feel music, which not only affects individual physiological indicators, but also has a certain effect on psychological indicators such as affects and cognitive state (Chair et al., 2021). Music is the most direct catalyst for triggering affective changes, and many studies have shown that it can effectively regulate affects and have a positive effect on improving bad moods, thus further establishing the importance of music in affect regulation research (Koelsch, 2010; Zanders, 2018; Silva et al., 2021). Participation in sports promotes physical health, mutual interaction and communication, psychological stress release, and emotion regulation, and it reduces depression, anxiety, and stress levels (Edwards et al., 2018). Research evidence suggests that human affects improve significantly after a one-time exercise, such as cycling, running, and endurance training (Focht et al., 2015; Fuss et al., 2015). This improvement is reflected in the emotional experience known as “runner’s high” after a running exercise (Hallgren, et al., 2010; Heggelund et al., 2014), contributing to positive affect enhancement and stress reduction. Moreover, reduced stress minimizes the negative emotional experiences of individuals (Hopkins et al., 2012; Hogan et al., 2013; Henneghan et al., 2020).

Related studies have also shown that the auditory stimulation of music at different exercise times positively impacts human physiology and psychology. Music improves personal affect and reduces subjective fatigue during moderate-intensity exercise (Innocenti et al., 2022). The benefits of combining movement and music are not only observed during training, but music as a therapeutic aid also positively contributes to recovery from post-exercise fatigue (Karageorghis et al., 2009). Studies have shown that listening to music can reduce the rating of perceived exercise (RPE) and subjective fatigue among exercisers (Karageorghis et al., 2006a). An extensive search of the empirical research literature reported internationally has revealed that most exercise–music interventions aimed at improving human mental health or real-time emotions combine “exercise” and “music” simultaneously or have focused on a single-phase activity during the time of auditory music stimulation. However, studies are rare in terms of comparing the effects of music auditory interventions on human mental health improvement or real-time affect at different times of the exercise process (e.g., before, during, and after exercise or throughout the exercise process). Aiming to address the research gap, this study adopts a randomized controlled trial design, giving the same auditory music stimuli before, during, and after aerobic power cycling and throughout the exercise process, to compare the effect of each group on affect improvement. The findings of this research can provide a reference for future combined exercise–music intervention strategies.

## 3 Research methods

### 3.1 Participants

A total of 140 students were recruited, and 120 of them were enrolled in this experiment. The admission criteria were as follows: 1) the age of the subjects must be between 19 and 30 years old; 2) a physical activity adaptation questionnaire (Physical Activity Readiness Questionnaire, PARQ) must confirm the ability of the subjects to participate in moderate-intensity aerobic exercise; and 3) the subjects had not participated in similar studies in the past. Exclusion criteria for subjects were as follows: color blindness, infectious diseases and meeting the requirements of the PARQ questionnaire were excluded.

This experiment was approved by the Ethics Committee of Zhejiang Normal University (Approval No. ZSRT2022028), and the project was approved by the China Clinical Trial Registry (Registration No. ChiCTR2300068339). This research was funded by the National Social Science Foundation of China (Project No. 23FTYB007). All subjects were aware of the contents and requirements of this study, and they signed informed consent forms. Prior to the experiment, the participants were given two reminders: 1) not to participate in strenuous physical activities, such as ball games, aerobics, mountain climbing, and other joint activities, during the first 3 days of the experiment and 2) not to drink beverage products, such as coffee, alcohol, and tea, not to eat spicy and irritating food during the first 3 days of the experiment.

Furthermore, before the experiment, the proportion of sampling loss was thoroughly considered. The target participants were college students, and 140 of them were recruited based on the questionnaire requirements. The participants were randomly divided into four music intervention groups, namely, the pre-exercise, during-exercise, post-exercise, and whole-course groups. In the initial stage, this study recorded abnormal signal reception by a heart rate band. Consequently, 11 participants were not able to complete the experiment, while 9 participants had missing data after the data exportation. Overall, only 120 participants completed the survey.

**FIGURE 1:**
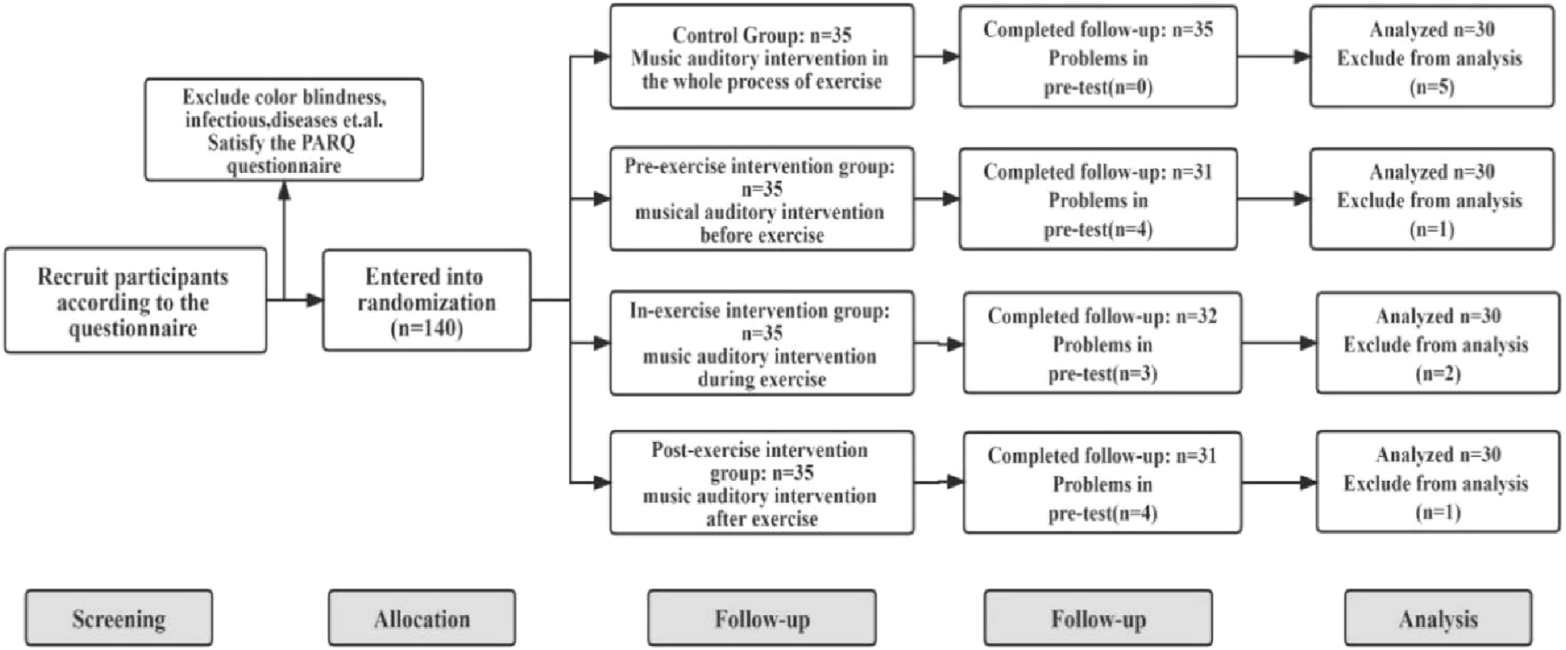
Flow chart of subject selection.

An independent team member not involved in other phases of the research project was given the task to randomly assign the participants. Each recruited participant was assigned a code. Then, after the baseline data collection, the participants were randomly assigned under experimental or control conditions. The team members responsible for randomization were blind to the situation of the participants. Meanwhile, the data collectors were blind to the grouping of participants throughout the study. The flowchart of the experimental allocation is shown in Figure 1.

After the effective screening, the total number of valid subjects who completed the experiment was 120. Their age range was 19–30 years old, with a mean age of 23.9 years old. The participants were composed of 60 males and 60 females, thus accounting for 50% of the valid issues. In terms of the educational level, one of the valid subjects was a first-year student (0.8%), seven were sophomores (5.8%), six were juniors (5.0%), four were seniors (3.3%), and 102 were postgraduates, including graduate students (85%). Table 1 shows the four experimental groups. Their differences were not statistically significant in terms of age, height, weight, and BMI.

**TABLE 1:** Participants demographic characteristics.

Group		Pre-exercise intervention	In-exercise intervention	Post-exercise intervention	Control group	*p*
N		30	30	30	30	
Age/years		23.63 ± 1.33	24.20 ± 2.34	24.13 ± 1.55	23.67 ± 1.45	0.433
Height/cm	Male	175.80 ± 4.30	178.40 ± 5.91	176.67 ± 4.80	176.80 ± 3.93	0.517
	Female	162.13 ± 3.98	164.93 ± 4.56	165.80 ± 4.72	165.33 ± 5.61	0.157
Weight/kg	Male	69.80 ± 6.86	74.80 ± 9.87	70.13 ± 8.96	72.33 ± 5.50	0.297
	Female	54.33 ± 10.83	54.93 ± 5.70	55.33 ± 3.33	55.00 ± 7.31	0.986
BMI/(kg·m^−2^)		21.47 ± 3.10	21.77 ± 2.45	21.30 ± 2.23	21.57 ± 2.30	0.911

### 3.2 Music program

Previous studies indicate that the music preferences of the subjects will likely affect their exercise, a phenomenon that has also been corroborated by the music habits of students. If issues pertaining to the choice of music are taken into consideration, then the critical variables of the experiment will vary from person to person (Koelsch, 2014; Li et al., 2019). The problem related to the lack of rigor among subjects due to their different music preferences was solved by allowing the researcher to choose the music rather than focusing on this preference issue. Meanwhile, to ensure consistency among the participant’s thematic preference, this study adopted the method of “being represented” by the participant. Thus, the most listened music list was viewed via NetEase Cloud Music by using the keyword “sports,” and the top-ranking song listened to by 1.41 million people was selected. A fast music with a tempo of 120–160 bpm was ultimately chosen. The selected musical characteristics are shown in Table 2.

**TABLE 2:** Characteristics of music.

Track	Duration (s)	Rhythm/bpm
Like I Would (Tom Budin Remix)	3 min42	125
The New Kings	3 min42	128
Energy Drink	5 min03	128
Temple	4 min55	124
River Flows In You (Original Mix)	4 min58	128
Lake Arrowhead (Radio Mix)	3 min35	122
First Of The Year	4 min18	145
Catch My Breath (Suprafive Remix)	4 min35	122
Don’t You Worry, Child	3 min39	129
Morsmordre	3 min37	124
Blame	3 min34	128
Remember Our Summer	2 min43	128
The Right Path	2 min28	126
Silver Scrapes	3 min19	140

### 3.3 Measurement indicators

#### 3.3.1 Heart rate variability

In the controlled combined exercise–music intervention experiments, the low frequency to high frequency (LF/HF) ratio is often used as the frequency domain analysis index (Zhou et al., 2010; Park et al., 2017; Mojtabavi et al., 2020). The LF/HF ratio can determine the equilibrium of sympathetic and vagal nerves or the modulation degree of the sympathetic nerves. The time domain analysis index primarily uses the root mean square of the difference between adjacent full RR intervals (RMSSD) and the standard deviation of continuous regular RR intervals (SDNN) as comprehensive reflective markers of the effect of short-intervention control experiment on affect improvement (Chen et al., 2020; Parizek et al., 2021). Therefore, LF/HF, RMSSD, and SDNN data were collected in this experimental study as the metrics for processing and analysis.

For the acquisition of the above-heart rate variability (HRV) indices, the First Beat Sports wireless physiological data collection system with the ECG module device was selected for the experiment. The apparatus can detect and capture changes in the subject’s heart rate in real time and during the activity. Simultaneously, the changing signal of the subject’s heart rate can be automatically converted into time- and frequency domain data in the background for recording and storage. The selected equipment has been used in a number of controlled experiments involving exercise in conjunction with music.The real-time accuracy and reliability of the data recorded by the aforementioned device system have been verified in many controlled experiments (Karageorghis et al., 2006b; Yamashita et al., 2006).

At the test site, the subjects were instructed to wear their heart rate belts correctly according to the requirements. Then, a background system would monitor the heart rate signal of a subject for successful matching, and the system would track and record data on real-time heart rate and the HRV indices. After the experiment, the HRV data recorded by the device during the pre-test,mid-test and post-test phases were extracted. Then, the LF/HF, RMSSD, and SDNN data were calculated according to the definitions of these three indices.

#### 3.3.2 Blood pressure

Two indicators, namely, diastolic blood pressure (DBP) and systolic blood pressure (SBP), were collected from the study sample. A decrease in either SBP or DBP indicates an improvement in the negative sexual affect. The BPs of the subjects were collected using the Ohm HEM-7121 portable electronic BP tester; the accuracy and reliability of the instrument for measuring BP indicators have been confirmed in relevant studies (Barton and Pretty, 2010; Liu et al., 2021). The BPs were collected in the inactive state, with the subject’s eyes looking in the direction of a specified field of view. After placing the arm of a subject on the table, with his/her elbow joint flushed with the heart position, the staff taking the BP ensured that the arm component of the subject before and after the test was consistent. In addition, interference with the staff’s manipulation movements was avoided by measuring the subject’s BP twice in a consecutive manner. The results of the two measurements were taken separately as average DBP and average SBP.

#### 3.3.3 Positive/negative affects

The “affective” index is an excellent method of recording and evaluating the real-time mental health status of subjects. In this experiment, the international instrument called the Positive/Negative Affect Scale (I-PANAS-SF short-volume version) was used to assess the subjects’ real-time emotional status.The validity of the Positive/Negative Emotions Scale was examined in detail in the findings of Thompson et al. The Cronbach’s alpha values for the I-PANAS-SF PA and NA subscales were 0.78 and 0.76, respectively, which indicated that the scale had good reliability and validity (Thompson, 2007). The reliability of this scale has been confirmed in many controlled experiments (Liu et al., 2012; Terry et al., 2020). The short-form version of the Positive/Negative Emotion Scale consists of ten questions, divided into five positive questions (quick-thinking, encouraged, determined, focused, and active states) and five negative questions (upset, hostile, shy, nervous, and afraid). During the pre-test and post-test phases, the subjects were asked to rate the positive and negative items according to their real-time emotional state. Finally, the total positive and negative scores were calculated separately to reflect the subjects’ emotional state for the pre-test and post-test.

### 3.4 Experimental process

**FIGURE 2:**
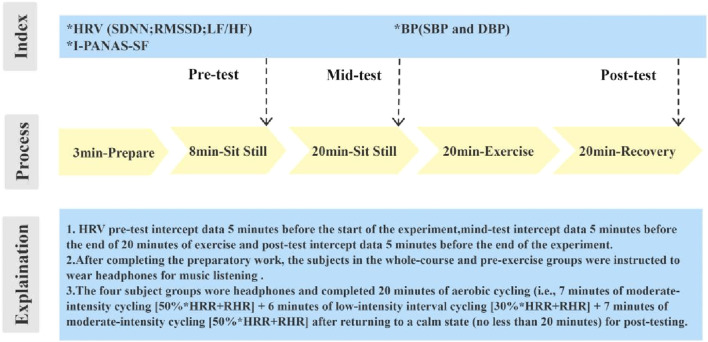
Experiment-specific flow chart.

Some studies have shown that exercising in the morning can improve mental status. According to these results, exercising in the morning not only activates metabolism and physiological activity, but also increases energy, which improves mental activity. In addition, sweating in the morning can improve mental health and productivity throughout the day, as exercise is helpful in reducing stress. However, there are also studies proving that evening exercise is equally beneficial in promoting health (Kalak et al., 2012; Irandoust et al., 2019). Since this experiment was conducted within a laboratory setting, to ensure the reliability and accuracy of the experimental data, data collection for all subjects was conducted at the same time period in the afternoon of the day, which was divided into the following two main time points, 14:30-15:30 and 15:30-16:30. The specific experimental flow chart is shown in Figure 2.

(1) **Preparation stage:** The experimenter prepared the appropriate experimental equipment for each group test. As soon as the subjects arrive at the practical test site, the experimenter introduces the experimental test instructions to them. The subjects were asked to read the Ethical Approval Letter authorized by the Academic Research Ethics Committee of Zhejiang Normal University and fill in the informed consent form for the experiment. Then, the informed consent of the subject was taken before starting the formal test. This phase took about 3 min.
(2) **Pre-testing phase:** Before the physical activity intervention, the individual demographic variables, daily physical activity levels, and different individual factor scales were collected. The dependent variables were gathered in the following order: a) The SBP and DBP were measured using an Omron electronic BP monitor. b) The First Beat wearable wireless physiological device was used to collect the HRV data 5 min before the start of the experiment. c) The subjects were asked to fill in the real-time “Positive Affect Scale” and “Negative Affect Scale.” This phase took about 8 min.
(3) **Intervention (mid-testing) phase:** After completing the preparatory work, the subjects in the whole-course and pre-exercise groups were instructed to wear headphones for music listening (laboratory sound intensity below 40 dB). After listening to the music for 20 min, they took BP measurements and answered the I-PANAS-SF scale. The collected indices were used as the mid-test data. The heart rate at this time was calculated as the target heart rate.

After completing the mid-test data collection, the subjects were asked to perform a moderate-intensity power bike ride (Swedish MONARK power bike) for 20 min. The specific activity process can be described as follows. First, the subjects were asked to perform the power bike ride at an activity intensity as a means of maintaining the target heart rate of about 50%*HRR + RHR for about 7 min. Then, the subjects were asked to perform power cycling activities to reach the target heart rate of about 30%*HRR + RHR for 6 min. Finally, the subjects were instructed to perform power cycling activities at a target heart rate of about 50%*HRR + RHR for 7 min.

(4) **Post-testing phase:** After the intervention, the heart rate band was taken off only when the subjects’ heart rate had recovered to the quiet state compared with that before the start of the experiment. The subjects’ recovery time should be at least 20 min, and their modest heart rate should be maintained for 30 s or more compared with that before the start of the experiment. Subsequently, the post-testing phase was immediately started. The order of data collection for the dependent variables in the post-testing phase was the same as that in the pre-testing stage. a) The SBP and DBP were measured using an Omron electronic BP monitor. b) The First Beat wearable wireless physiological device was used to collect the HRV data 5 min before the start of the experiment. c) The subjects were asked to fill in the real-time “Positive Affect Scale” and “Negative Affect Scale.” This phase took about 25 min.

**FIGURE 3:**
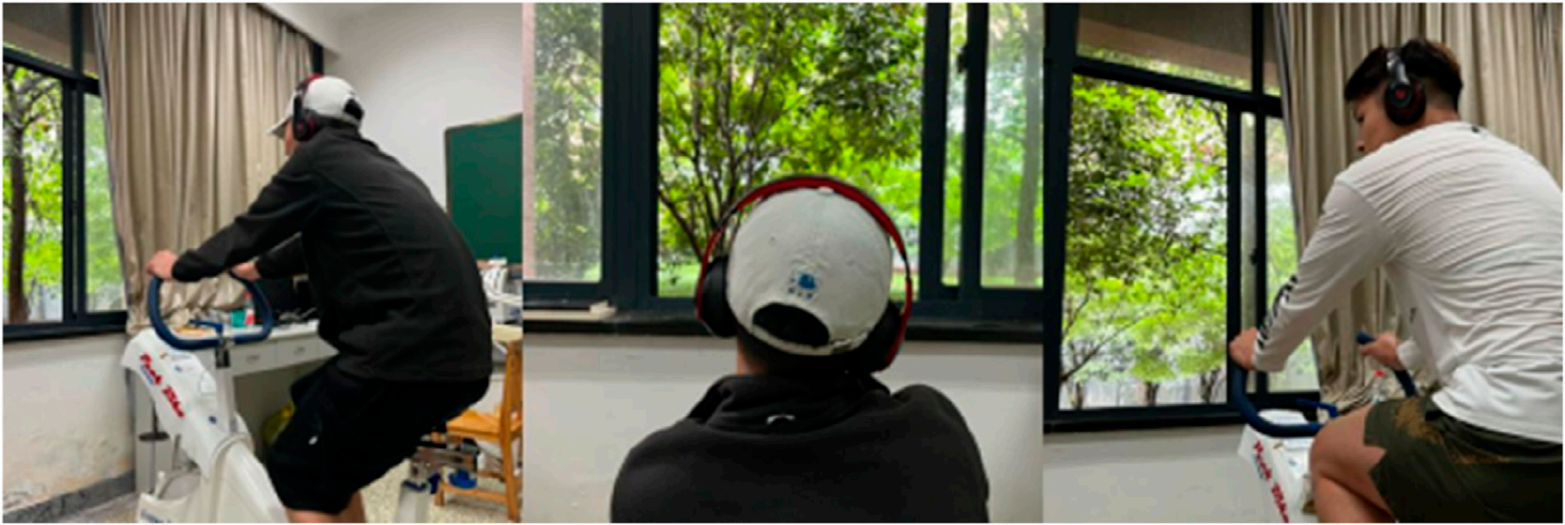
Experimental site photos.

After completing the physical activity intervention phase, the experimental staff provided the subjects with 500 mL of purified drinking water. The subjects were allowed to choose whether to consume the entire content or drink in moderation according to their conditions and needs. To avoid unnecessary interference with the HRV and other indicators during the recovery phase, the subjects were prohibited from drinking pure water during the recovery phase after the initial drinking. Photographs of the experimental site are shown in Figure 3.

## 4 Results

### 4.1 Comparison of the variability of the dependent variable pre-test, mid-test, and post-test

#### 4.1.1 BP indices

**FIGURE 4:**
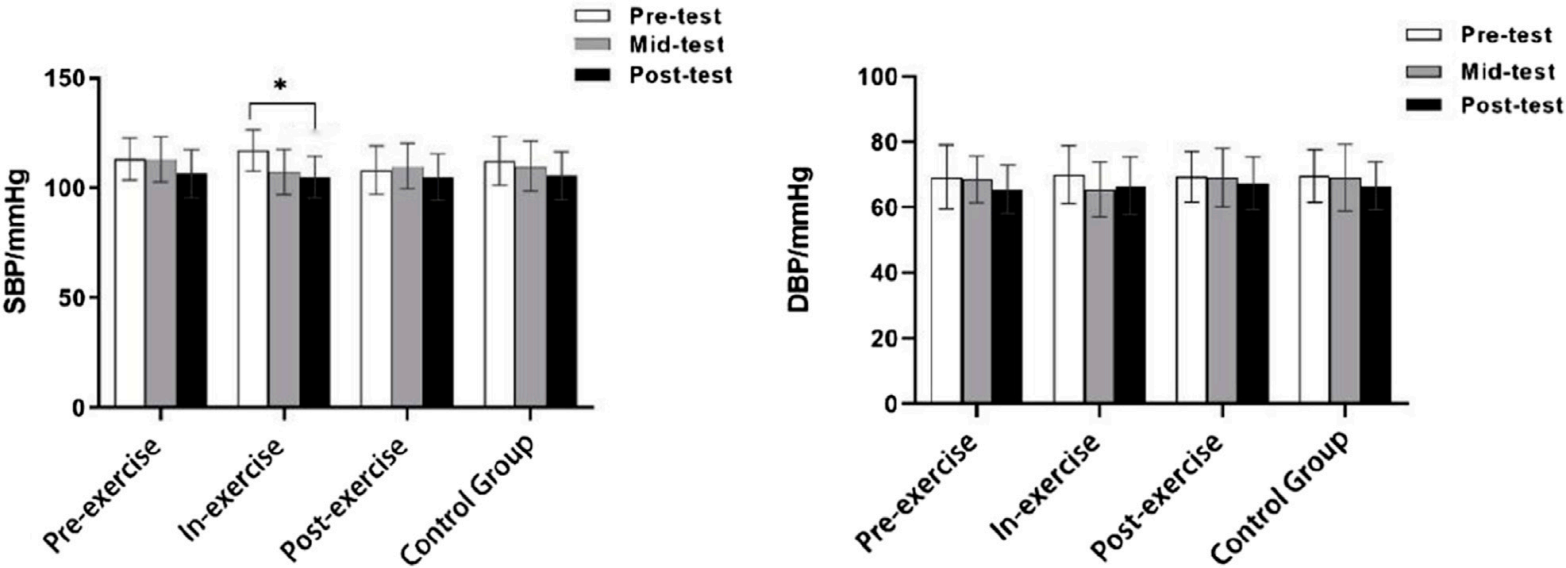
The trend of pre-test and post-test changes in systolic and diastolic blood pressure.

No significant differences were observed between the changes in SBP and DBP indices in the pre-exercise group in the pre-, mid-, and post-tests (*F* = 4.078, *p* = 0.20, *ɳp* ^*2*^ = 0.086; *F* = 1.748, *p* = 0.180, *ɳp* ^*2*^ = 0.039; Table 3). In the during-exercise group, the subjects’ SBP decreased from 111.17 ± 9.38 mmHg on the pre-test to 107.20 ± 10.43 mmHg on the mid-test. The post-test value of SBP was 104.90 ± 9.41 mmHg, and significant differences in the SBP index were determined in the pre-, mid-, and post-tests in the during-exercise group (*F* = 3.170, *p* = 0.047, *ɳp* ^*2*^ = 0.068). Furthermore, no significant differences were observed in the changes in DBP indices in the pre-, mid-, and post-tests (*F* = 2.202, *p* = 0.117, *ɳp* ^*2*^ = 0.048). The differences were also non-significant between the changes in the pre- and post-tests of the SBP and DBP indices in the post-exercise group (*F* = 1.615, *p* = 0.205, *ɳp* ^*2*^ = 0.0364; *F* = 0.563, *p* = 0.572, *ɳp* ^*2*^ = 0.013). Moreover, no significant differences were determined for the SBP indices in the pre- and post-tests of the whole-course group (*F* = 1.405, *p* = 0.251, *ɳp* ^*2*^ = 0.031). Finally, the differences were non-significant for the changes in the DBP indices in the pre-, mid- and post-tests (*F* = 1.041, *p* = 0.358, *ɳp* ^*2*^ = 0.023). The trends of systolic and diastolic blood pressure before and after the test are shown in Figure 4.

**TABLE 3:** Difference test results of real-time emotional indicators in the control group and the experimental group.

	Pre-exercise		In-exercise		Post-exercise		Control group	
	M±SD	Multi-factor ANOVA	M±SD	Multi-factor ANOVA	M±SD	Multi-factor ANOVA	M±SD	Multi-factor ANOVA
Positive Affects								
Pre-test	6.87 ± 2.52	*F* = 2.672	6.73 ± 1.98	*F* = 5.516	7.53 ± 2.87	*F* = 0.220	7.20 ± 2.91	*F* = 5.053
Mid-test	5.97 ± 1.33	*p* = 0.075	5.93 ± 1.20*#	*p* = 0.006	7.30 ± 2.35	*p* = 0.803	6.80 ± 2.04#	*p* = 0.008
Post-test	5.83 ± 1.60*	*ɳp* ^2^ = 0.058	5.50 ± 1.01***	*ɳp* ^*2*^ = 0.113	7.10 ± 2.34	*ɳp* ^*2*^ = 0.005	5.53 ± 0.90**	*ɳp* ^*2*^ = 0.104
Negative Affects								
Pre-test	12.93 ± 4.09	*F* = 0.791	12.97 ± 2.92	*F* = 5.135	14.03 ± 3.07	*F* = 0.660	13.27 ± 3.29	*F* = 2.720
Mid-test	14.30 ± 4.19*	*p* = 0.457	16.10 ± 4.29**#	*p* = 0.008	15.00 ± 4.03	*p* = 0.519	15.40 ± 4.44**	*p* = 0.071
Post-test	13.63 ± 4.34	*ɳp* ^*2*^ = 0.018	14.90 ± 4.11**	*ɳp* ^*2*^ = 0.106	15.03 ± 4.29	*ɳp* ^*2*^ = 0.015	14.97 ± 3.40**	*ɳp* ^*2*^ = 0.059

Note: The above models are designed as follows: intercept + gender + age + education level. (*) indicates a significant difference between the mid-test and post-test, respectively, and the pre-test, were *: *p* < 0.05; **: *p* < 0.01; ***: *p* < 0.001. (#) indicates a significant difference between the mid-test and post-test, where #: *p* < 0.05.

#### 4.1.2 Positive or negative affect

**FIGURE 5:**
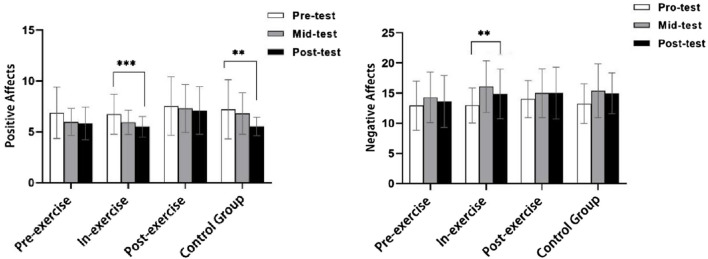
Pre-test, mid-test, and post-test trends of positive and negative emotions.

As shown in Table 4, in the pre-exercise group, the overall trend of the subjects’ negative affect scores slightly decreases in the pre- and post-tests, but the differences are not statistically significant (*F* = 2.672, *p* = 0.075, *ɳp* ^*2*^ = 0.058). Meanwhile, the subjects’ positive affect scores in the pre- and post-tests showed an overall trend of an increase followed by a decrease, but the differences are not significant (*F* = 0.791, *p* = 0.457, *ɳp* ^*2*^ = 0.018). In the during-exercise group, an overall decreasing trend was determined for the subjects’ negative affect, and the changes in the pre- and post-tests were significantly different (*F* = 5.516, *p* = 0.006, *ɳp* ^*2*^ = 0.113). In addition, the subjects’ positive affect scores on the pre- and post-tests showed an overall trend of an increase followed by a decrease, and these changes were significantly different (*F* = 5.135, *p* = 0.008, *ɳp* ^*2*^ = 0.106). In the post-exercise group, the overall trend slightly decreased, but no significant differences were determined in the pre- and post-tests (*F* = 0.220, *p* = 0.803, *ɳp* ^*2*^ = 0.005). In addition, the subjects’ positive affect scores were 14.03 ± 3.07 on the pre-test, 15.00 ± 4.03 on the mid-test, and 15.03 ± 4.29 on the post-test, showing a slight overall upward trend, and the difference was non-significant between the pre- and mid-tests for this indicator (*F* = 0.660, *p* = 0.519, *ɳp* ^*2*^ = 0.015). In the whole-course group, the subjects showed significant differences in the changes in the pre- and post-test values for the negative affect (*F* = 5.053, *p* = 0.008, *ɳp* ^*2*^ = 0.104). In addition, the overall trend of the subjects’ positive affective scores on the pre- and post-tests increased and then decreased, but no significant differences were observed in the changes for this index (*F* = 2.720, *p* = 0.071, *ɳp* ^*2*^ = 0.059). Pre-test, mid-test and post-test trends for positive and negative emotions are shown in Figure 5.

**TABLE 4:** The difference test results of the central rate variability index between the control group and the experimental group.

	Pre-exercise		In-exercise		Post-exercise		Control group	
	M±SD	Multi-factor ANOVA	M±SD	Multi-factor ANOVA	M±SD	Multi-factor ANOVA	M±SD	Multi-factor ANOVA
HRV-LF/HF								
Pre-test	2.65 ± 1.82	*F* = 3.451	2.48 ± 1.53	*F* = 5.698	2.35 ± 1.32	*F* = 6.825	2.55 ± 1.58	*F* = 3.509
Mid-test	4.04 ± 2.23	*p* = 0.036	4.51 ± 2.90	*p* = 0.005	4.25 ± 2.67	*p* = 0.002	4.12 ± 2.69	*p* = 0.034
Post-test	3.39 ± 2.09**	*ɳp* ^2^ = 0.074	3.69 ± 2.39**	*ɳp* ^*2*^ = 0.116	3.65 ± 1.89*	*ɳp* ^*2*^ = 0.136	4.02 ± 3.18**	*ɳp* ^2^ = 0.075
HRV-SDNN [ms]								
Pre-test	64.89 ± 24.75	*F* = 13.078	60.34 ± 20.52	*F* = 18.994	67.65 ± 26.14	*F* = 16.100	62.05 ± 18.48	*F* = 23.861
Mid-test	35.71 ± 19.26*#	*p* = 0.032	30.09 ± 19.17	*p* = 0.412	34.66 ± 20.42	*p* = 0.044	30.30 ± 19.38	*p* = 0.026
Post-test	57.91 ± 24.77**	*ɳp* ^*2*^ = 0.231	50.42 ± 18.38	*ɳp* ^*2*^ = 0.304	56.14 ± 21.61**	*ɳp* ^*2*^ = 0.270	51.96 ± 16.60*	*ɳp* ^*2*^ = 0.354
HRV-RMSSD [ms]								
Pre-test	65.08 ± 28.91	*F* = 23.680	63.32 ± 24.66	*F* = 29.912	67.68 ± 31.56	*F* = 17.088	59.60 ± 26.98	*F* = 29.406
Mid-test	29.28 ± 8.03	*p* = 0.001	29.09 ± 6.04	*p* = 0.032	33.50 ± 11.75**#	*p* = 0.002	24.95 ± 6.01	*p* = 0.043
Post-test	51.16 ± 18.37**	*ɳp* ^*2*^ = 0.352	53.16 ± 16.88*	*ɳp* ^*2*^ = 0.407	53.11 ± 20.38**	*ɳp* ^*2*^ = 0.282	50.56 ± 14.99*	*ɳp* ^*2*^ = 0.403

Note: The above models are designed as follows: intercept + gender + age + education level. (*) indicates a significant difference between the mid-test and post-test, respectively, and the pre-test, were *: *p* < 0.05; **: *p* < 0.01; ***: *p* < 0.001. (#) indicates a significant difference between the mid-test and post-test, where #: *p* < 0.05.

#### 4.1.3 HRV indices

**FIGURE 6:**
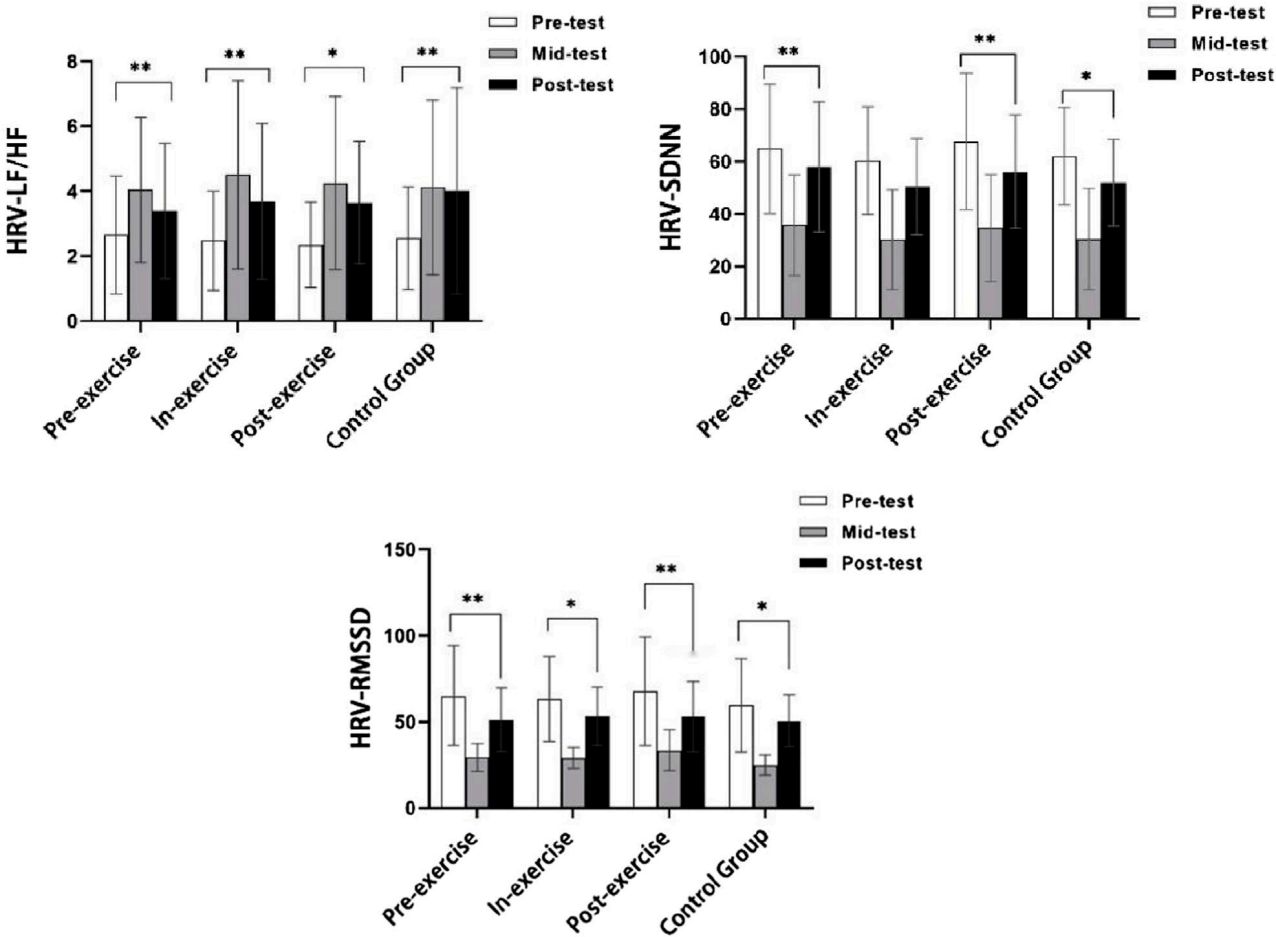
The trend of pre-test, mid-test, and post-test of SDNN, RMSSD, and LF/HF.

As shown in Table 5, in the pre-exercise group, the differences are significant in terms of the changes in LF/HF values in the pre-, mid- and post-tests (*F* = 3.451, *p* = 0.036, *ɳp* ^*2*^ = 0.074). In addition, the SDNN values showed a trend of a decrease followed by an increase in the pre- and post-tests, and the differences in the changes were significant (*F* = 13.078, *p* = 0.032, *ɳp* ^*2*^ = 0.231). The same significant differences were observed for the changes in the subjects’ RMSSD in the pre-, mid- and post-tests (*F* = 23.680, *p* = 0.001, *ɳp* ^*2*^ = 0.352). In the during-exercise group, significant differences were observed in the changes in LF/HF in the pre-, mid-, and post-tests (*F* = 5.698, *p* = 0.005, *ɳp* ^*2*^ = 0.116). The SDNN values showed a trend of a decrease followed by an increase in the pre-and post-tests, but a significant difference was not reached (*F* = 18.994, *p* = 0.0412, *ɳp* ^*2*^ = 0.304). In addition, significant differences were observed in the changes in the pre- and mid-, post-tests of the RMSSD indicators in this group (*F* = 29.912, *p* = 0.032, *ɳp* ^*2*^ = 0.407). In the post-exercise group, significant differences were observed for the change in the LF/HF values of the subjects in the pre- and post-tests (*F* = 6.825, *p* = 0.002, *ɳp* ^*2*^ = 0.136). The SDNN values of the samples showed a trend of a decrease and followed by an increase in the pre- and post-tests, and the changes reached a significant difference (*F* = 16.100, *p* = 0.044, *ɳp* ^*2*^ = 0.270). As shown in Table 5 substantial differences are depicted for the changes in the subjects’ RMSSD values in the pre- and post-tests (*F* = 17.088, *p* = 0.002, *ɳp* ^*2*^ = 0.282). In the whole-course group, the differences were significant for the changes in the pre-, mid-, and post-tests of the LF/HF values of the subjects (*F* = 3.509, *p* = 0.034, *ɳp* ^*2*^ = 0.075). The SDNN values of showed a trend of a decrease followed by an increase in the pre-, mid-, and post-tests, and the changes were significantly different (*F* = 23.861, *p* = 0.026, *ɳp* ^*2*^ = 0.354). Moreover, significant differences were observed in the changes in the subjects’ RMSSD values in the pre- and post-tests (*F* = 29.406, *p* = 0.043, *ɳp* ^*2*^ = 0.403). The trends of SDNN, RMSSD and LF/HF before, during and after the test are shown in Figure 6.

**TABLE 5:** Effect of music intervention at different periods on differences in changes in blood pressure indicators.

	Pre-exercise	In-exercise	Post-exercise	Control group	Comparison between groups
SBP [mmHg]					
Pre-test	113.33 ± 9.59	111.17 ± 9.38	108.13 ± 11.10	112.37 ± 11.03	*F* = 2.379
Mid-test	113.03 ± 10.22	107.20 ± 10.43	110.00 ± 10.26	109.83 ± 11.39	*p* = 0.030**
Post-test	106.50 ± 11.06	104.90 ± 9.41	105.10 ± 10.61	105.67 ± 10.98	*ɳp* ^*2*^ = 0.058
Comparison within groups	*F* = 4.078	*F* = 3.170	*F* = 1.615	*F* = 1.405	
	*p* = 0.20	*p* = 0.047	*p* = 0.205	*p* = 0.251	
	*ɳp* ^*2*^ = 0.086	*ɳp* ^*2*^ = 0.068	*ɳp* ^*2*^ = 0.036	*ɳp* ^*2*^ = 0.031	
DBP [mmHg]					
Pre-test	69.23 ± 9.79	70.00 ± 8.79	69.40 ± 7.75	69.63 ± 8.09	*F* = 1.729
Mid-test	68.60 ± 7.11	65.50 ± 8.4	69.17 ± 8.95	69.03 ± 10.18	*p* = 0.115
Post-test	65.53 ± 7.44	66.60 ± 8.77	67.33 ± 8.04	66.60 ± 7.36	*ɳp* ^*2*^ = 0.043
Comparison within groups	*F* = 1.748	*F* = 2.202	*F* = 0.563	*F* = 1.041	
	*p* = 0.180	*p* = 0.117	*p* = 0.572	*p* = 0.358	
	*ɳp* ^*2*^ = 0.039	*ɳp* ^*2*^ = 0.048	*ɳp* ^*2*^ = 0.013	*ɳp* ^*2*^ = 0.023	

Note: Gender, age, and education level were adjusted for the above models based on repeated measures ANOVA, test.

### 4.2 Comparison of the variability of the dependent variables in groups with different exercise–music interventions

#### 4.2.1 BP indices at different periods of music intervention

As shown in Table 6, the differences in the effects of music intervention on SBP (*F* = 2.379, *p* = 0.030, *ɳp* ^*2*^ = 0.058) at different periods were significant. Comparative results indicate that the during-exercise group (*F* = 3.170, *p* = 0.047, *ɳp* ^*2*^ = 0.068) had better SBP improvement than the pre-exercise group (*F* = 4.078, *p* = 0.20, *ɳp* ^*2*^ = 0.086), post-exercise group (*F* = 1.615, *p* = 0.205, *ɳp* ^*2*^ = 0.036), and whole-course group (*F* = 1.405, *p* = 0.251, *ɳp* ^*2*^ = 0.031). This finding suggests that music intervention during exercise contributes more to the reduction of SBP than at other times. However, comparative results of the effect of DBP was not substantial across the four groups (*F* = 1.729, *p* = 0.115, *ɳp* ^*2*^ = 0.043).

**TABLE 6:** Effect of music interventions at different periods on differences in changes in real-time effects indicators.

	Pre-exercise	In-exercise	Post-exercise	Control group	Comparison between groups
Positive Affects					
Pre-test	6.87 ± 2.52	6.73 ± 1.98	7.53 ± 2.87	7.20 ± 2.91	*F* = 1.599
Mid-test	5.97 ± 1.33	5.93 ± 1.20	7.30 ± 2.35	6.80 ± 2.04	*p* = 0.148
Post-test	5.83 ± 1.60	5.50 ± 1.01	7.10 ± 2.34	5.53 ± 0.90	*ɳp* ^*2*^ = 0.040
Comparison within groups	*F* = 2.672	*F* = 5.516	*F* = 0.220	*F* = 5.053	
	*p* = 0.075	*p* = 0.006	*p* = 0.803	*p* = 0.008	
	*ɳp* ^*2*^ = 0.058	*ɳp* ^*2*^ = 0.113	*ɳp* ^*2*^ = 0.005	*ɳp* ^*2*^ = 0.104	
Negative Affects					
Pre-test	12.93 ± 4.09	12.97 ± 2.92	14.03 ± 3.07	13.27 ± 3.29	*F* = 2.451
Mid-test	14.30 ± 4.19	16.10 ± 4.29	15.00 ± 4.03	15.40 ± 4.44	*p* = 0.043**
Post-test	13.63 ± 4.34	14.90 ± 4.11	15.03 ± 4.29	14.97 ± 3.40	*ɳp* ^*2*^ = 0.091
Comparison within groups	*F* = 0.791	*F* = 5.135	*F* = 0.660	*F* = 2.720	
	*p* = 0.457	*p* = 0.008	*p* = 0.519	*p* = 0.071	
	*ɳp* ^*2*^ = 0.018	*ɳp* ^*2*^ = 0.106	*ɳp* ^*2*^ = 0.015	*ɳp* ^*2*^ = 0.059	

Note: Gender, age, and education level were adjusted for the above models based on repeated measures ANOVA, test.

#### 4.2.2 Real-time affect indicators at different periods of music intervention

Table 7 shows a significant difference in the effect of music intervention on the positive affect scores in all groups (*F* = 2.451, *p* = 0.043, *ɳp* ^*2*^ = 0.091). Nonetheless, comparative results indicate that the effect of music intervention on the during-exercise group (*F* = 5.135, *p* = 0.008, *ɳp* ^*2*^ = 0.106) was better than those on the other three groups in terms of improving the subjects’ positive emotions. This finding implies that music intervention during exercise is more helpful in enhancing individuals’ positive emotions compared with other times. However, the results did not show significant differences in the effect of music intervention on negative affect in all groups (*F* = 1.599, *p* = 0.148, *ɳp* ^*2*^ = 0.040).

**TABLE 7:** Effect of music intervention at different periods on the difference in the change of heart rate variability index**.**

	Pre-exercise	In-exercise	Post-exercise	Control group	Comparison between groups
*HRV-LF/HF*					
Pre-test	2.65 ± 1.82	2.48 ± 1.53	2.35 ± 1.32	2.55 ± 1.58	*F* = 0.134
Mid-test	4.04 ± 2.23	4.51 ± 2.90	4.25 ± 2.67	4.12 ± 2.69	*p* = 0.940
Post-test	3.39 ± 2.09	3.69 ± 2.39	3.65 ± 1.89	4.02 ± 3.18	*ɳp* ^*2*^ = 0.003
Comparison within groups	*F* = 3.451	*F* = 5.698	*F* = 6.825	*F* = 3.509	
	*p* = 0.036	*p* = 0.005	*p* = 0.002	*p* = 0.034	
	*ɳp* ^*2*^ = 0.074	*ɳp* ^*2*^ = 0.116	*ɳp* ^*2*^ = 0.136	*ɳp* ^*2*^ = 0.075	
*HRV-SDNN [ms]*					
Pre-test	64.89 ± 24.75	60.34 ± 20.52	67.65 ± 26.14	62.05 ± 18.48	*F* = 1.167
Mid-test	35.71 ± 19.26	30.09 ± 19.17	34.66 ± 20.42	30.30 ± 19.38	*p* = 0.325
Post-test	57.91 ± 24.77	50.42 ± 18.38	56.14 ± 21.61	51.96 ± 16.60	*ɳp* ^*2*^ = 0.029
Comparison within groups	*F* = 13.078	*F* = 18.994	*F* = 16.100	*F* = 23.861	
	*p* = 0.032	*p* = 0.412	*p* = 0.044	*p* = 0.026	
	*ɳp* ^*2*^ = 0.231	*ɳp* ^*2*^ = 0.304	*ɳp* ^*2*^ = 0.270	*ɳp* ^*2*^ = 0.354	
*HRV-RMSSD [ms]*					
Pre-test	65.08 ± 28.91	63.32 ± 24.66	67.68 ± 31.56	59.60 ± 26.98	*F* = 0.961
Mid-test	29.28 ± 8.03	29.09 ± 6.04	33.50 ± 11.75	24.95 ± 6.01	*p* = 0.414
Post-test	51.16 ± 18.37	53.16 ± 16.88	53.11 ± 20.38	50.56 ± 14.99	*ɳp* ^*2*^ = 0.024
Comparison within groups	*F* = 23.680	*F* = 29.912	*F* = 17.088	*F* = 29.406	
	*p* = 0.001	*p* = 0.032	*p* = 0.002	*p* = 0.043	
	*ɳp* ^*2*^ = 0.352	*ɳp* ^*2*^ = 0.407	*ɳp* ^*2*^ = 0.282	*ɳp* ^*2*^ = 0.403	

Note: Gender, age, and education level were adjusted for the above models based on repeated measures ANOVA test.

#### 4.2.3 HRV indices at different periods of music intervention

Table 8 shows the comparative results of the time domain indicators related to HRV of the four groups, including the time domain effects of SDNN (*F* = 1.167, *p* = 0.325, *ɳp* ^*2*^ = 0.029) and RMSSD (*F* = 0.961, *p* = 0.414, *ɳp* ^*2*^ = 0.024). None of the differences reached a significant level. The difference between the effects of the groups on the frequency domain index of LF/HF was also not significant (*F* = 0.134, *p* = 0.940, *ɳp* ^*2*^ = 0.003).

**TABLE 8:** Effect of music intervention at different periods on the difference in the change of heart rate variability index.

	Pre-exercise	In-exercise	Post-exercise	Control group	Comparison between groups
HRV-LF/HF					
Pre-test	2.65 ± 1.82	2.48 ± 1.53	2.35 ± 1.32	2.55 ± 1.58	*F* = 0.134
Mid-test	4.04 ± 2.23	4.51 ± 2.90	4.25 ± 2.67	4.12 ± 2.69	*p* = 0.940
Post-test	3.39 ± 2.09	3.69 ± 2.39	3.65 ± 1.89	4.02 ± 3.18	*ɳp* ^*2*^ = 0.003
Comparison within groups	*F* = 3.451	*F* = 5.698	*F* = 6.825	*F* = 3.509	
	*p* = 0.036	*p* = 0.005	*p* = 0.002	*p* = 0.034	
	*ɳp* ^*2*^ = 0.074	*ɳp* ^*2*^ = 0.116	*ɳp* ^*2*^ = 0.136	*ɳp* ^*2*^ = 0.075	
HRV-SDNN [ms]					
Pre-test	64.89 ± 24.75	60.34 ± 20.52	67.65 ± 26.14	62.05 ± 18.48	*F* = 1.167
Mid-test	35.71 ± 19.26	30.09 ± 19.17	34.66 ± 20.42	30.30 ± 19.38	*p* = 0.325
Post-test	57.91 ± 24.77	50.42 ± 18.38	56.14 ± 21.61	51.96 ± 16.60	*ɳp* ^*2*^ = 0.029
Comparison within groups	*F* = 13.078	*F* = 18.994	*F* = 16.100	*F* = 23.861	
	*p* = 0.032	*p* = 0.412	*p* = 0.044	*p* = 0.026	
	*ɳp* ^*2*^ = 0.231	*ɳp* ^*2*^ = 0.304	*ɳp* ^*2*^ = 0.270	*ɳp* ^*2*^ = 0.354	
HRV-RMSSD [ms]					
Pre-test	65.08 ± 28.91	63.32 ± 24.66	67.68 ± 31.56	59.60 ± 26.98	*F* = 0.961
Mid-test	29.28 ± 8.03	29.09 ± 6.04	33.50 ± 11.75	24.95 ± 6.01	*p* = 0.414
Post-test	51.16 ± 18.37	53.16 ± 16.88	53.11 ± 20.38	50.56 ± 14.99	*ɳp* ^*2*^ = 0.024
Comparison within groups	*F* = 23.680	*F* = 29.912	*F* = 17.088	*F* = 29.406	
	*p* = 0.001	*p* = 0.032	*p* = 0.002	*p* = 0.043	
	*ɳp* ^*2*^ = 0.352	*ɳp* ^*2*^ = 0.407	*ɳp* ^*2*^ = 0.282	*ɳp* ^*2*^ = 0.403	

Note: Gender, age, and education level were adjusted for the above models based on repeated measures ANOVA, test.

## 5 Discussion

### 5.1 Improvement of the subjects’ affect owing to the combined exercise–auditory music stimulation

In this experimental study, subjective and objective assessment indices were combined to determine the affective improvement effect of the subjects before and after music intervention. The impact on the affect improvement was measured and evaluated comprehensively to achieve the complementary exercise–music intervention advantages with strong objectivity and accuracy. The subjective assessment of the positive/negative affect has been used and validated in related studies. Furthermore, music therapy has been determined to have excellent psychological modulating effects, as it relieves tension of individuals and improves their mental health through empathy, suggestion, and guidance; it also reduces pain and improves language and cognitive abilities in people with dementia.

Neuropsychological studies have demonstrated that music has a direct effect on the human neural structure, especially on the cerebral cortex, with its ability to increase the release of endorphins in the body and alter catecholamine levels in the body (Koelsch et al., 2016; Rodriguez et al., 2021), thereby relieving pain and reducing blood pressure, heart rate, respiratory rate, oxygen consumption and serum lactate levels (De Witte et al., 2020; de Andrade et al., 2022). Scherer and Zentner (2001) emphasized that music may affect us by triggering emotional associations, a process that may rely on subcortical mechanisms (Scherer and Zentner, 2001). The theoretical framework proposed by Juslin (2013) suggests eight psychological mechanisms that include brainstem reflexes, rhythmic traction, evaluative conditioning, contagion, visual imagery, situational memory, musical anticipation, and aesthetic judgment as eight psychological mechanisms by which music influences emotional responses (Juslin, 2013). Among other things, brainstem reflexes refer to the process by which the fundamental acoustic properties of music stimulate a response by signaling a potentially important or urgent event. For example, fast-paced music automatically stimulates listeners by activating the central nervous system, and this stimulation leads to elevated heart rate, blood pressure, body temperature, skin conductance, and muscle tone (Chapados and Levitin, 2008). While slow music produces the opposite effect, thus reducing sympathetic arousal (Chanda and Levitin, 2013). In addition, music therapy lowers the heart rate and BP and enhances the body’s immune function. The use of music at different exercise periods specifically affect people’s physiology and psychology.

Exercise combined with musical stimulation can improve the performance of individuals and help them improve their endurance and strength. The mechanisms why music stimulation can prolong exercise time can be summarized as follows. First, music can reduce the fatigue of individuals and improve their mental health to alleviate their subjective feelings of fatigue, hence the prolonged exercise time.Second, music is pleasantly stimulating and can block the upward transmission of physical stimulus response, thus prolonging exercise time. Music can be transmitted to the body through auditory organs where subtle and harmonious synchronized vibrations occur, and it can increase the excitability of nerve cells in the cerebral cortex, thus effectively improving personal affect. Furthermore, music stimulates the cortical nerve cells by mimicking the exact brain circuitry, thus increasing the neurotransmitter dopamine that positively improves the negative affect (Pilutti et al., 2013; Peretz, 2016; de Andrade et al., 2022).

The results of the study showed that the post-test scores of the subjects’ negative emotions have a decreasing trend compared with the pre-test scores after completing a certain degree of exercise intensity combined with music listening, and this trend is apparent in the four groups with different intervention periods. Moreover, the post-test scores of the subjects’ positive affect showed an increasing trend compared with the pre-test scores. Notably, in the post-exercise group, the subjects’ negative affect showed a decreasing trend. Nonetheless, the pre- and post-tests did not differ significantly. The decrease in the negative affect scores and the increase in the positive affect scores reflect the subjects’ tendency to improve their affect before and after the intervention experiment. In addition, music (120–140 bpm) combined with moderate-intensity aerobic exercise is more likely to improve the individuals’ affect states. The study of music intervention for acute exercise indicates that individuals who listen to music while exercising benefit from the enhancing affect. Musical stimuli act on the limbic system and brainstem reticular formation via auditory transmission. The modulation of music processing centers in the cerebral cortex positively affects human physiological functions and psychological states. The positive effect of aerobic exercise on human affect has also been previously established. At the same time, music is transmitted to the brain through the auditory nerve. Music frequency is somewhat symmetrical to brain frequency, producing a specific resonance, which, together with the notion of its synchronized state with exercise speed, brings a more positive emotional state to individuals (Wei et al., 2018; Schroder and Mackenzie, 2022). In fact, listening to music can increase exercise intensity by releasing higher levels of dopamine in the brain. Dopamine is a neurotransmitter associated with pleasure and motivation. Listening to music can also improve emotional states by increasing neural activity in the area of the brain that releases dopamine. This area of the brain is activated when an athlete decides to continue training despite fatigue, which is known as the explanatory model of training adjustment (McMorris et al., 2018).

In summary, exercise combined with fast-paced music not only can relieve physical fatigue caused by activity but also effectively improve the negative affect of exercise. In this study, the decrease in the subjects’ SBP and DBP reflects their mental averaging and relaxation during the exercise combined with auditory music stimulation, further suggesting that the effect on the subjects’ affect improvement can be facilitated. The values of the time domain indicators (RMSSD and SDNN) in all four groups showed a decreasing trend in the post-test phase compared with the pre-test phase. This scenario reflects the sympathetic nerve activity of the subjects, which were found to increase in the post-test phase of the intervention activity, indicating the increase in positive emotions of the subjects. In addition, LF/HF domain indicators in the post-test showed an increasing trend compared with those in the pre-test phase values. This difference indicates that the balance of autonomic modulation in the human body is biased in favor of sympathetic nerves after the exercise with the music intervention, consequently increasing the sympathetic activity. For the HRV index, the combination of frequency and time domain analyses reveal the tendency of the sympathetic nerve activity to dominate the end phase of the intervention prior to returning to a calm state, suggesting a certain degree of improvement in the subjects’ positive affect.

### 5.2 Analysis of the variability of different music intervention periods on the dependent variable

Terry and Karageorghis presented a model in 2006 to describe the benefits of physical activity accompanied by appropriate music (Karageorghis et al., 2006a). Although the positive effects of music on personal feelings may not alter the perception of fatigue during high-intensity exercise, music may change a person’s interpretation of or response to the perception of high-intensity activity. In other words, while music may not distract exercisers from the fatigue caused by high-intensity exercise, it may change their perception of that fatigue towards a more positive evaluation, thereby increasing their positive affect.The effect of listening to music during exercise-on-exercise performance may be influenced by music preference.

Most of the studies have shown that the results of the biorhythm theory can be used to improve people’s athletic performance, individual’s athletic performance depends on many factors, if the coaches or those who are in contact with the athletes are aware of the days when individuals are in the positive zone, the negative zone, or the key days of the body cycle, they can be more effective in making precise and appropriate scheduling of the exercises used for the physical aspects of exercise, which can help to improve the performance of the exercise as well as preventing the injuries of the exercise (Zareian et al., 2014). Moldovan et al. (2011) studied the performance of gymnasts and their biorhythmic cycle, the results of this study in both the positive and negative phases of the biorhythmic cycle showed that the subjects performed better in the positive phase (Moldovan et al., 2011). In addition, biological rhythms are equally important in the process of exercise combined with music interventions. In 1890, the German physician Wilhelm Fries discovered the existence of biological rhythmic cycles in the human body. He conducted extensive research on the state of his patients and found that they had a 23-day physiological cycle and a 28-day emotional cycle. According to the theory of biorhythms, every individual passes through life in regular harmony from birth to death, and their behavioral patterns follow three physical, emotional, and intellectual cycles, which begin according to an individual’s exact date of birth (Sha’bani Bahar et al., 2013). Therefore, focusing on an individual’s biorhythmic cycle could help to develop more precise exercise programs for exercisers in future studies.

The effect of the different periods of music intervention on the differences in the changes in the indicators of each dependent variable was further examined using a statistical model entailing repeated-measures ANOVA. The analysis revealed a significant difference in the effect of different intervention periods with respect to the change in the subjects’ negative affectivity in the four groups. In contrast to the findings for the pre-exercise, post-exercise and whole-course group, the effect on the subjects in the during-exercise group was more significant. This finding indicates that listening to music during exercise can significantly improve the negative emotions in humans. In addition, the effect of music on the during-exercise group was also significantly better than those of the other three groups in terms of positive affect scores.The values of the frequency domain (LF/HF) and time domain (SDNN and RMSSD) of the HRV index did not reach the significant level for the four groups in terms of physical activity intervention. The study results of the combined exercise–music intervention involving the four groups at different periods under the same exercise intensity showed that when subjects return to the calm state after completing a certain period of aerobic power cycling, the SBP and DBP in the post-test phase are lower than those in the pre-test phase in all four groups. The results showed a decreasing trend, and significant differences between the post-test and pre-test were established. In addition, a substantial difference was observed in the effect of the during-exercise group on SBP, indicating that musical auditory intervention during exercise contributes better in reducing SBP compared with the remaining three intervention periods.

In summary, in the during-exercise group, the subjects’ negativity affect scores improved better than those in the pre-exercise, post-exercise, and complete-course groups, and the differences were significant. In contrast to the results of the above indicators, the three indicators (SDNN, RMSSD, and LF/HF) showed somewhat better improvement in the post-exercise group, and the effects of the four different intervention periods on the differences in the changes in these indicators all reached the significant level.

### 5.3 Limitations

(1) The present study responded to the effect of affects improvement primarily through observations of blood pressure, positive/negative affect, and heart rate variability metrics, and the control of many of the covariates in this study was primarily exploratory, so future research may focus on conducting experiments using psychological and physiological measures appropriate for this area of research to more fully understand the effects of exercise combined with music on the effects of affects improvement in individuals in order to advance this area of research.
(2) Since all of our subjects were college students at Zhejiang Normal University and the age of the subjects was 19–30 years old, resulting in the results of this study may not be generalizable to other populations, consideration needs to be given to including subjects of different age groups in future studies to verify the generalizability of the results. In addition, studies conducted within a laboratory setting may limit the generalizability of the findings to real-world situations, and future research designs should be considered in real-world exercise situations to emphasize the generalizability of the findings.
(3) The study selected only popular music as the experimental material. A study showed that listening to music with a tempo of 100–180 bpm during joint exercise music can reduce the RPE rating during exercise; listening to music with a tempo of 140 bpm can significantly reduce exercise fatigue; and using music with a tempo of 140–160 bpm can effectively enhance athletic performance (Hutchinson and Karageorghis, 2013; Zijlema et al., 2018). Therefore, the effect of moderate-intensity aerobic exercise and listening to different types of music on affect improvement must be further evaluated. In addition, in this study, each group was divided equally between males and females. No gender differences were found in the statistical analysis; therefore, gender differences were not reported in the study.
(4) The results of previous studies have shown that there are differences in individual responses to music and exercise (Levitin et al., 2018; Zelechowska, 2020), and the results of our previous experiments did not show inter-individual differences, which is largely due to the small sample size, so the future study proposes to further expand the sample in order to carry out a more in-depth study to observe the improvement effect of moderate aerobic exercise combined with music on individuals’ affect.

## 6 Conclusion

(1) The degree of changes in SBP and DBP, HRV (SDNN, RMSSD, and LF/HF), and subjective affect indicators of the combined exercise–music intervention at different periods showed improvements in the subjects’ affect before and after music listening.The results of established studies have shown that prolonged exercise combined with music has a similarly significant improvement in the affects of individuals (Kampragkou et al., 2017; Satoh et al., 2020). Thus, exercise combined with music listening has a specific promotional effect on human affect improvement. This research suggests that combining music with physical activity is more helpful in improving human affect.In the context of this study, we use the term affects to refer to a neurophysiological state that is consciously acquired as a simple, primal feeling. While the effects on affects improvement in this study focused on short-term effects during and after exercise, we are also interested in the long-term effects of exercise combined with music interventions and plan to continue exploring this in future studies.
(2) Under the same intensity of aerobic power cycling exercise, the improvement effects of the emotional and physiological reflective indices (SDNN and RMSSD) and negative affect subjective rating indices were significantly better in the during-exercise group than those in the pre-exercise, post-exercise, and the whole-course groups. Meanwhile, the emotional and physiological reflective indices (SBP and DBP) and positive affect (LF/HF) were not significantly different in the four groups. No significant differences were observed among the four groups. This finding suggests that the effect of listening to music during exercise on affect improvement is better than in the other periods under the same activity pattern. This study recommends that when people perform physical activities, playing music during exercise is more helpful for promoting personal affect (Hutchinson and Karageorghis, 2013).

## Data Availability

The raw data supporting the conclusion of this article will be made available by the authors, without undue reservation.
